# Supplementary material for: Sevanol and Its Analogues: Chemical Synthesis, Biological Effects and Molecular Docking
Source: Pharmaceuticals (Basel). 2020 Jul 24;13(8):163. doi: 10.3390/ph13080163 (PMC7466040; doi:10.3390/ph13080163)

^{1}H NMR data for **1** in $CDCl_3$, 300MHz:

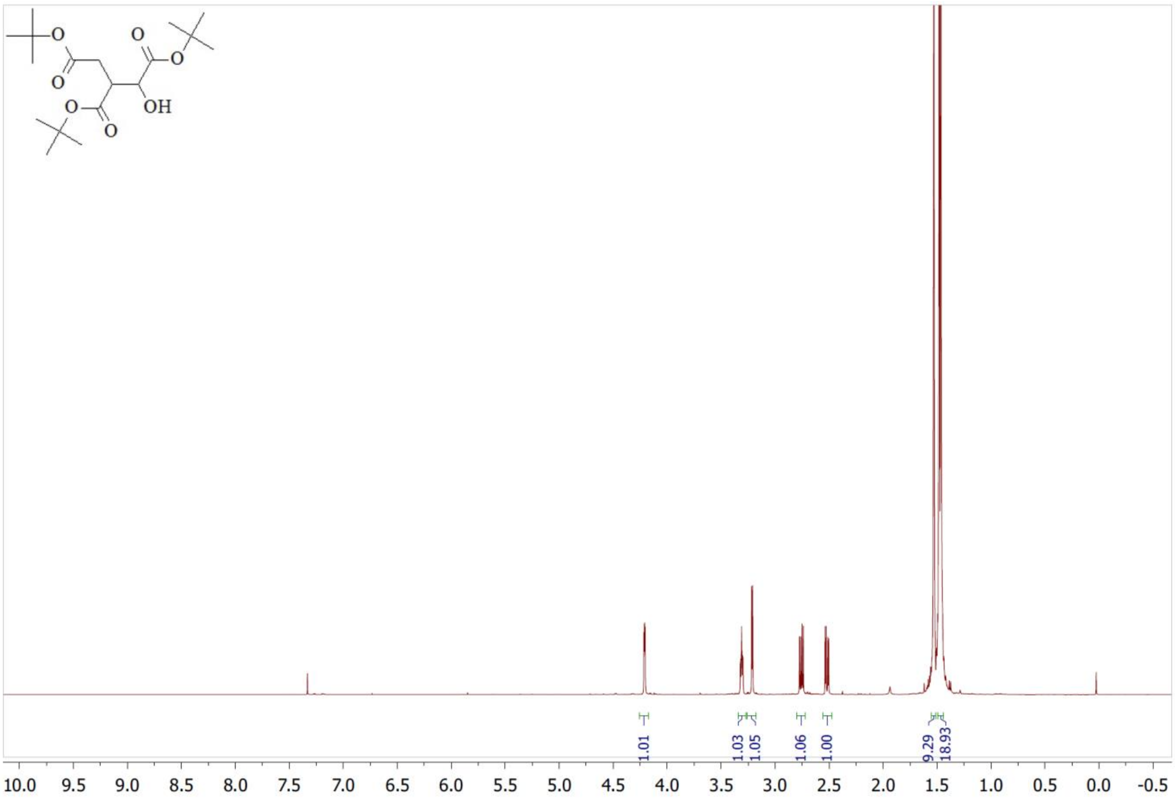

^{1}H and ^{13}C NMR data for **3b** in $CDCl_3$, 600MHz:

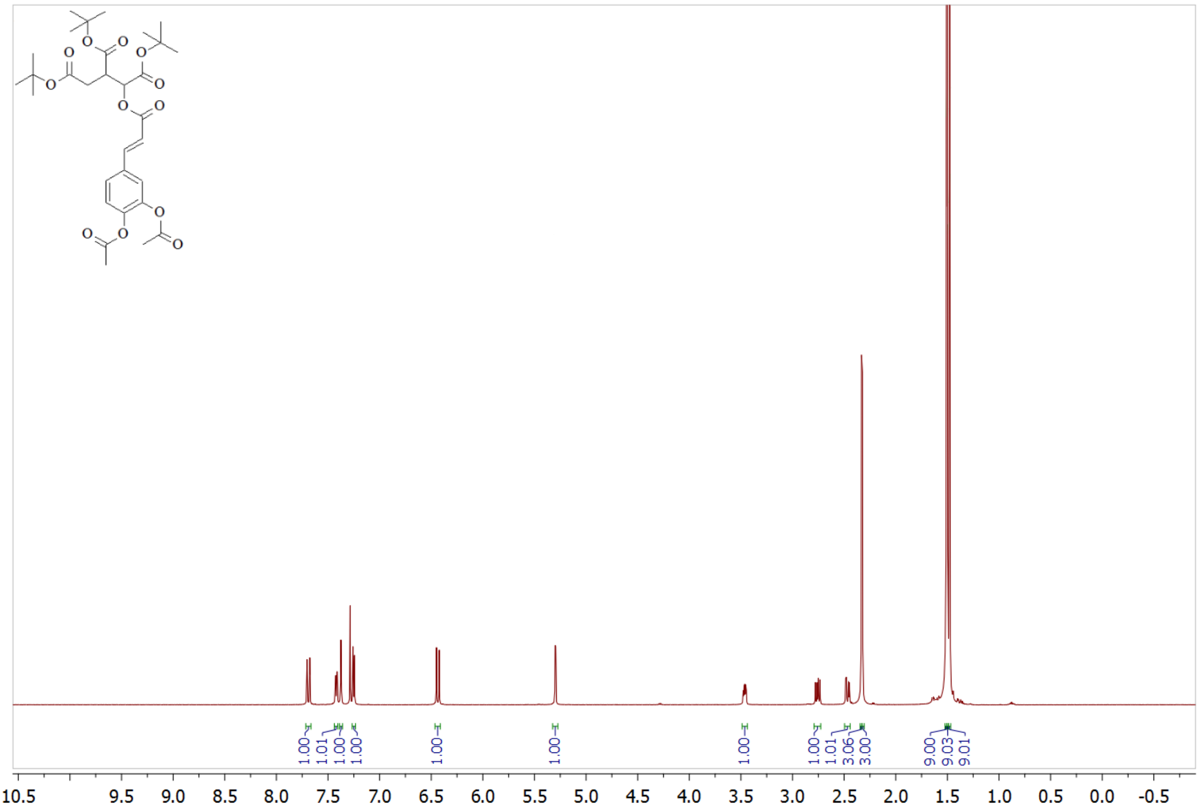

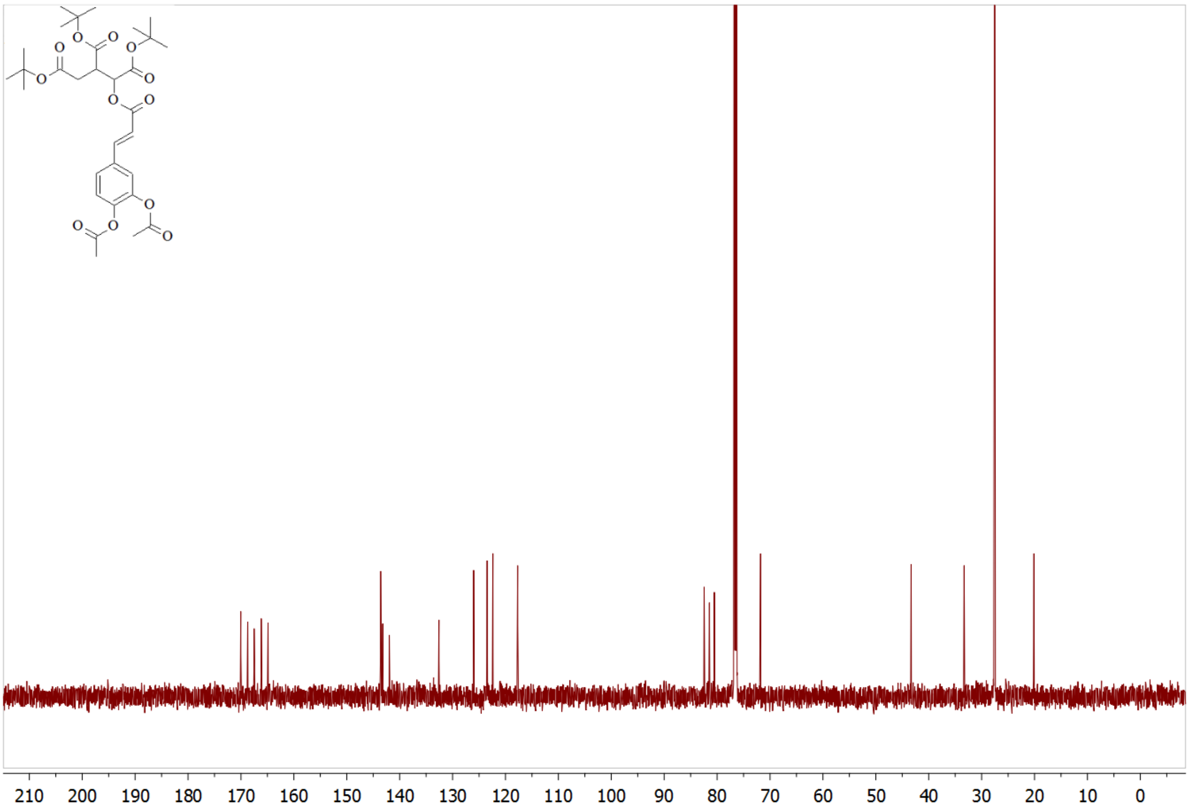

^{1}H and ^{13}C NMR data for **8** in $CDCl_3$, 600MHz:

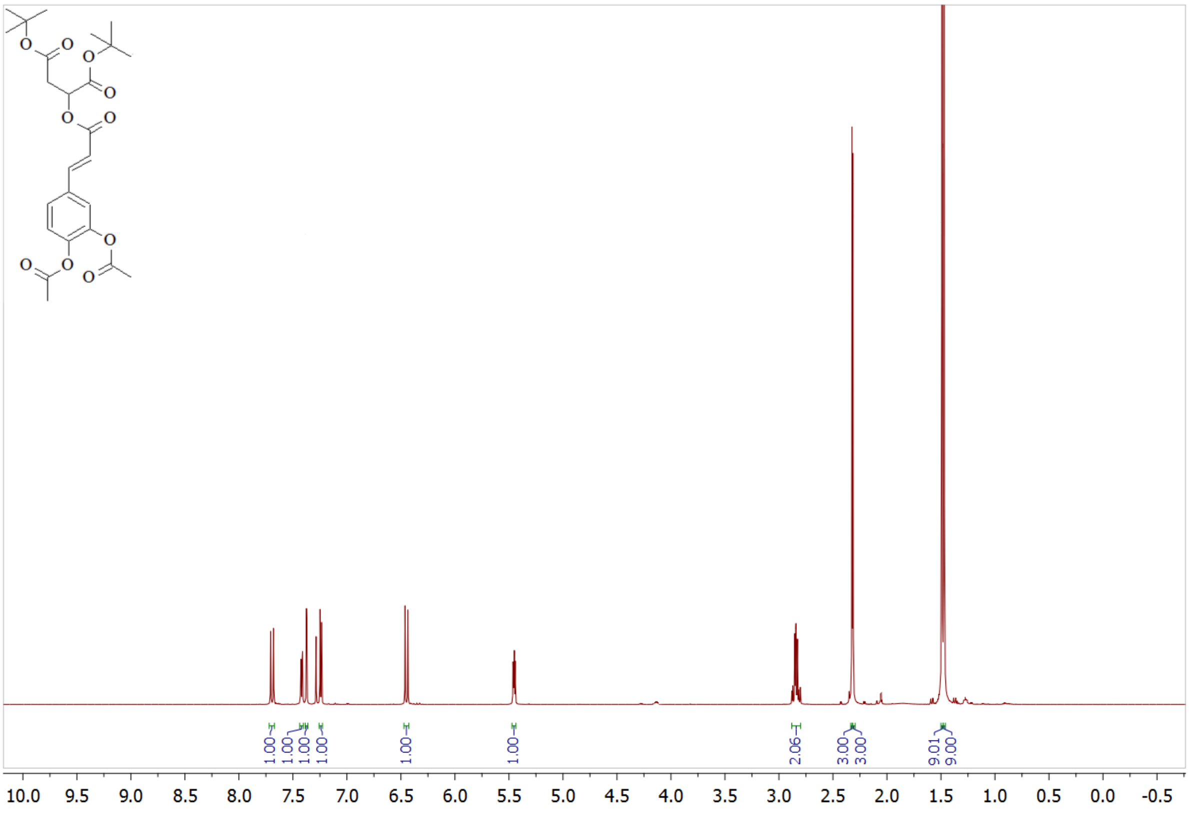

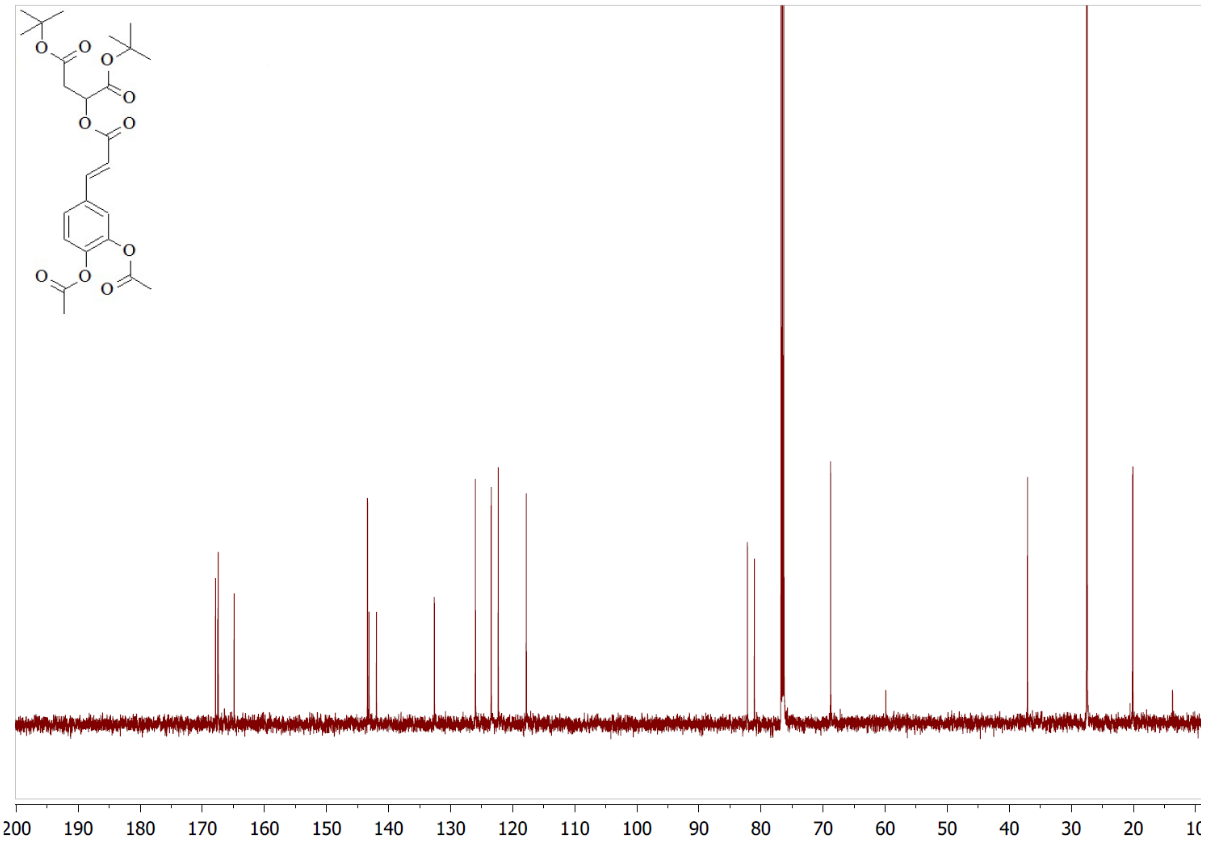

^{1}H NMR data for **4** in $CDCl_3$, 600MHz:

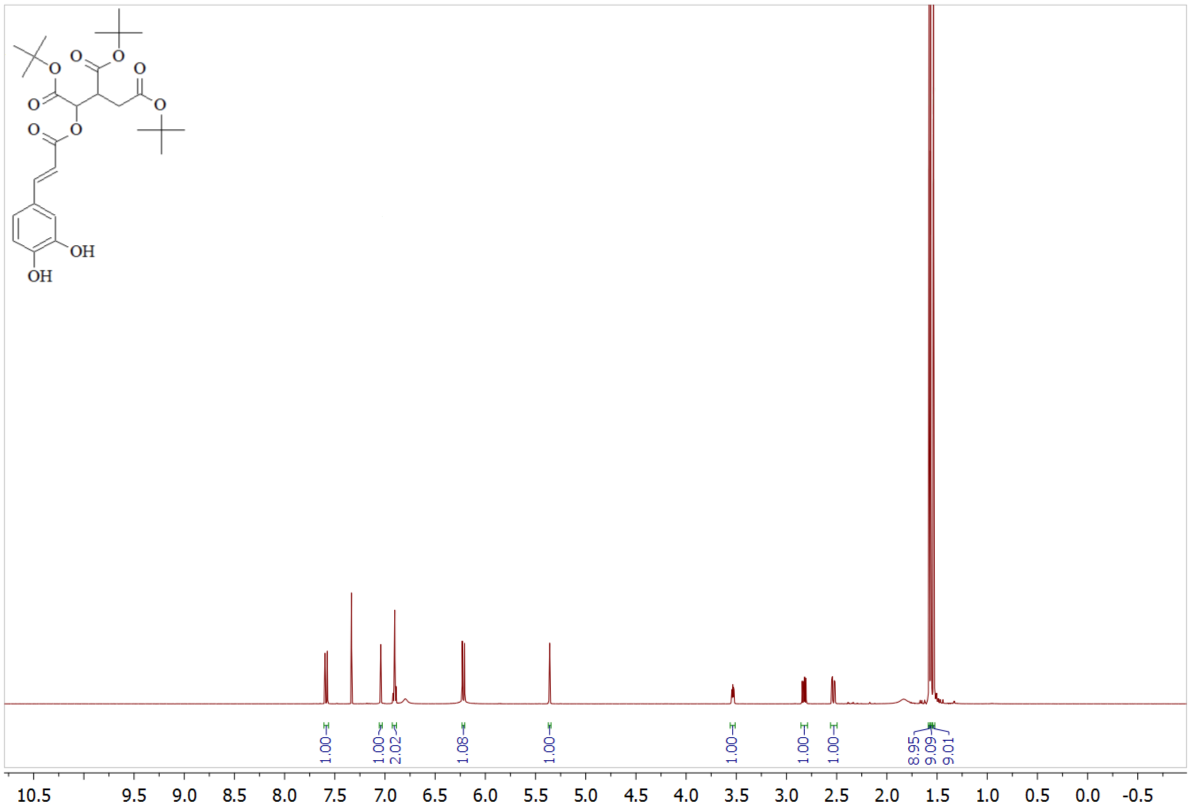

^{1}H and ^{13}C NMR data for **9** in $CDCl_3$, 600MHz:

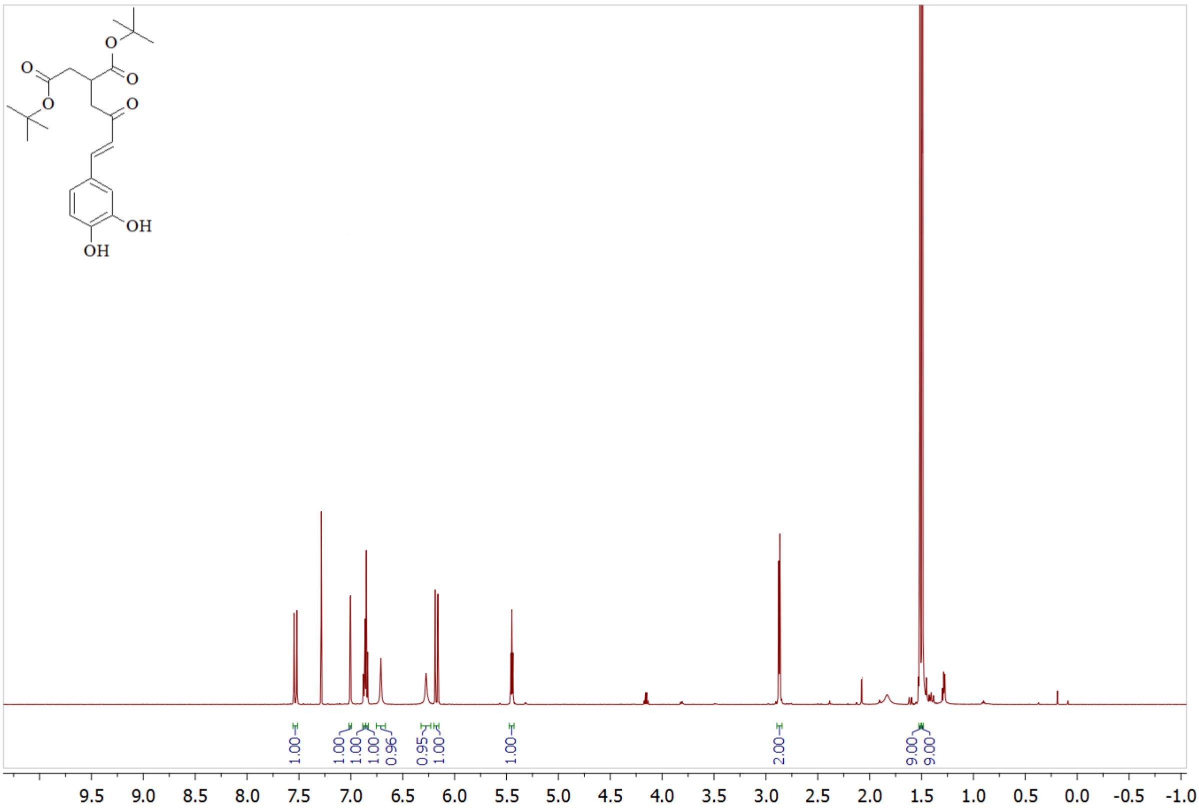

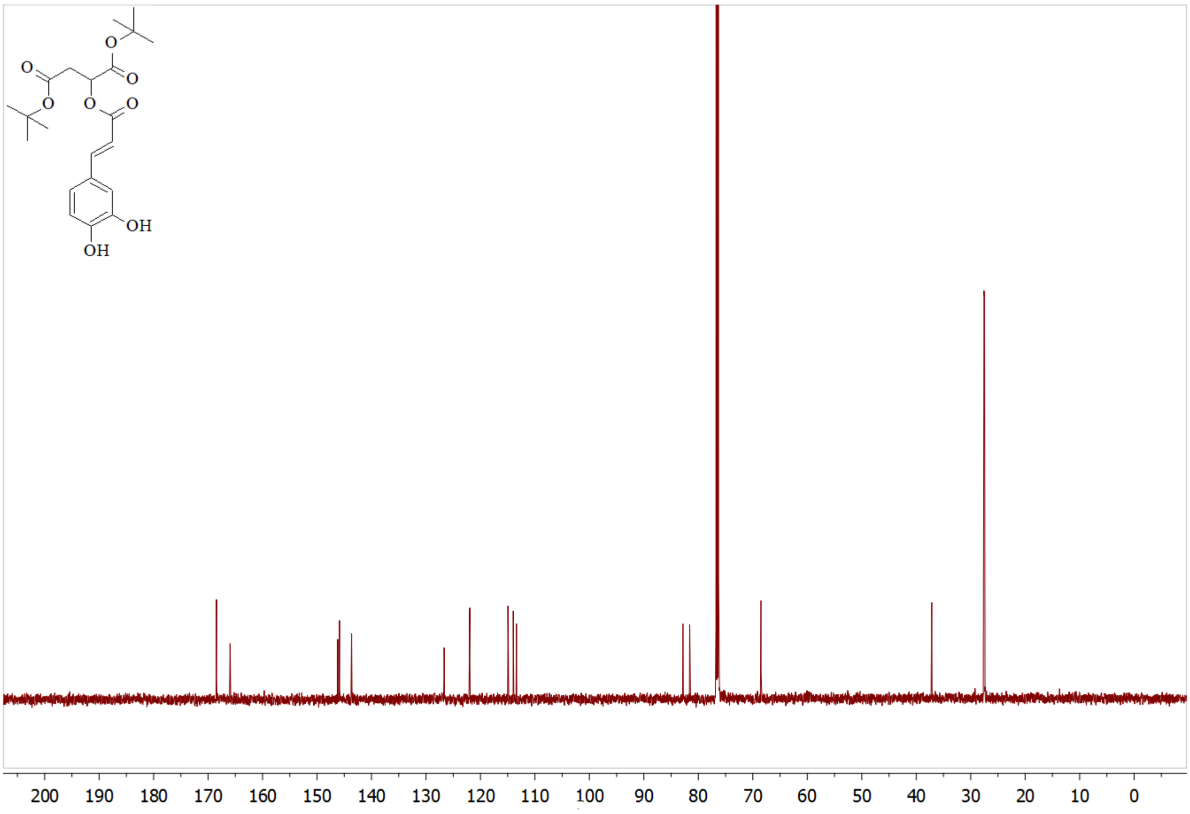

^{1}H and ^{13}C NMR data for **11** in acetone-d_6, 600MHz:

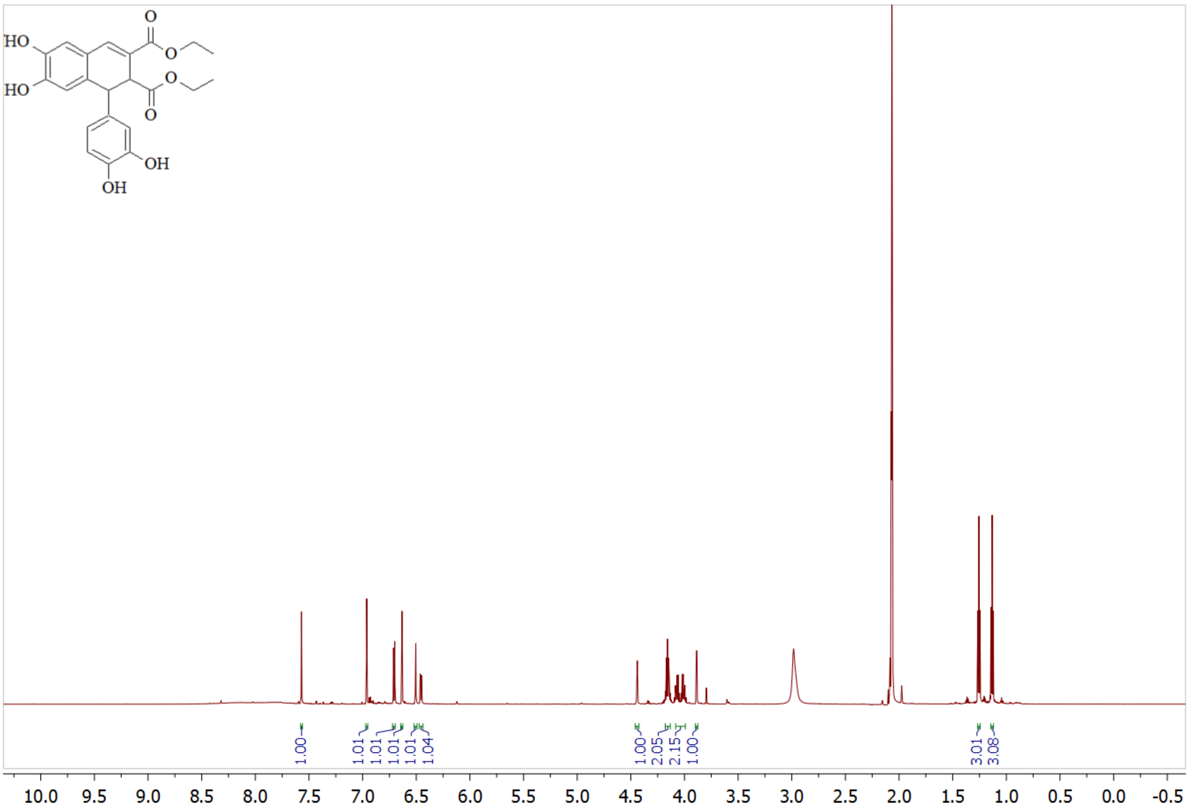

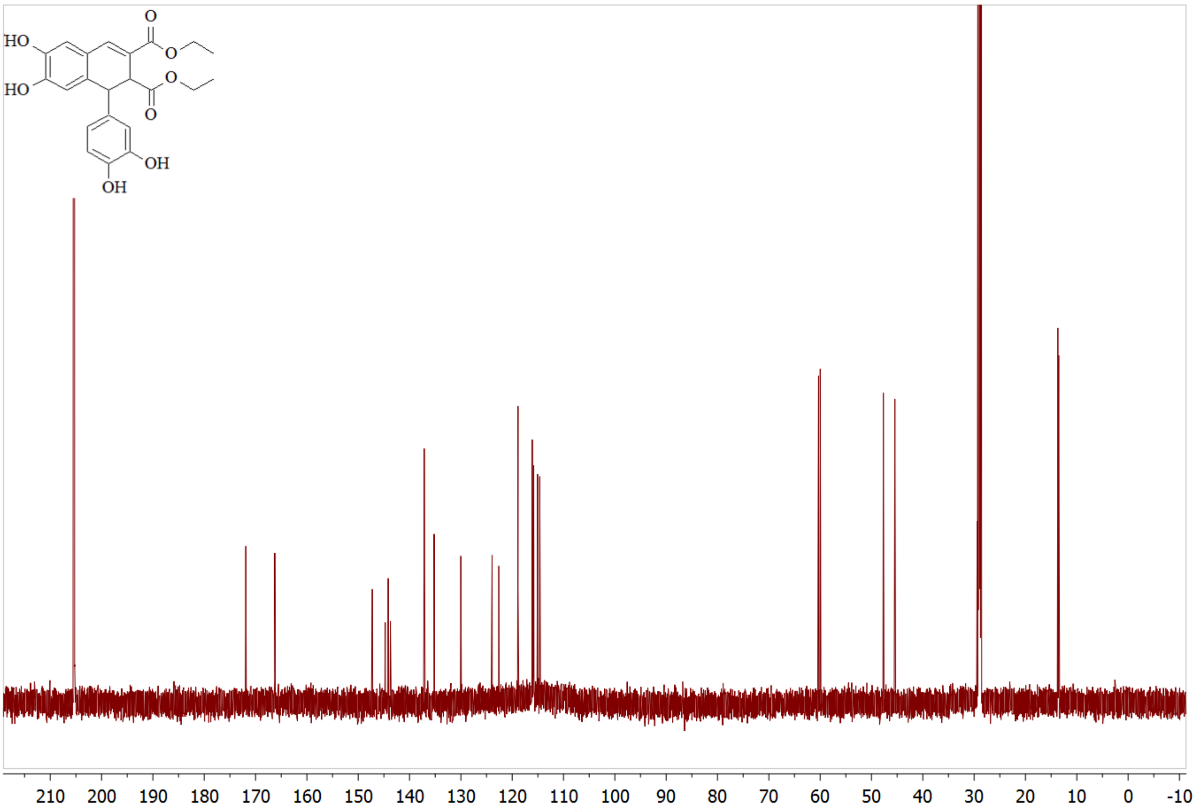

^1H and ^{13}C NMR data for **I (s706)** in ratio 1:3 (isosevanol:sevanol) in D_2O, 600MHz:

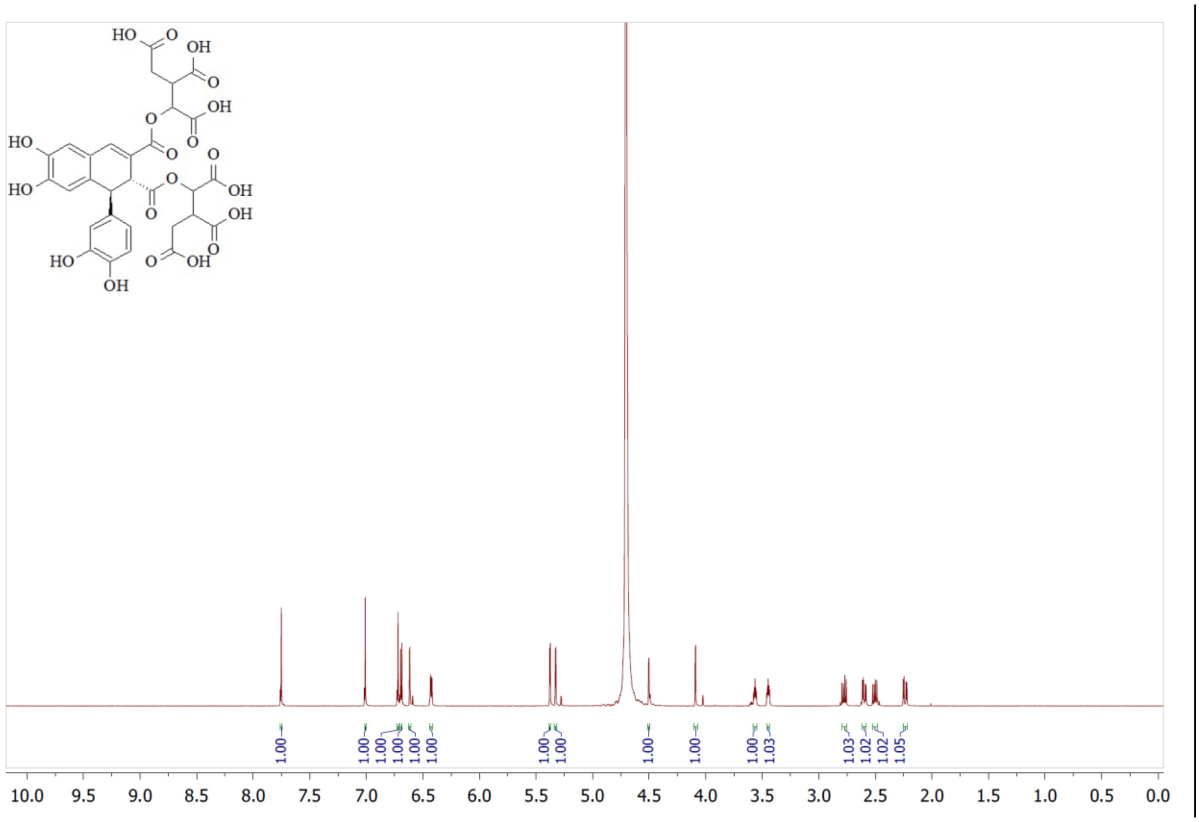

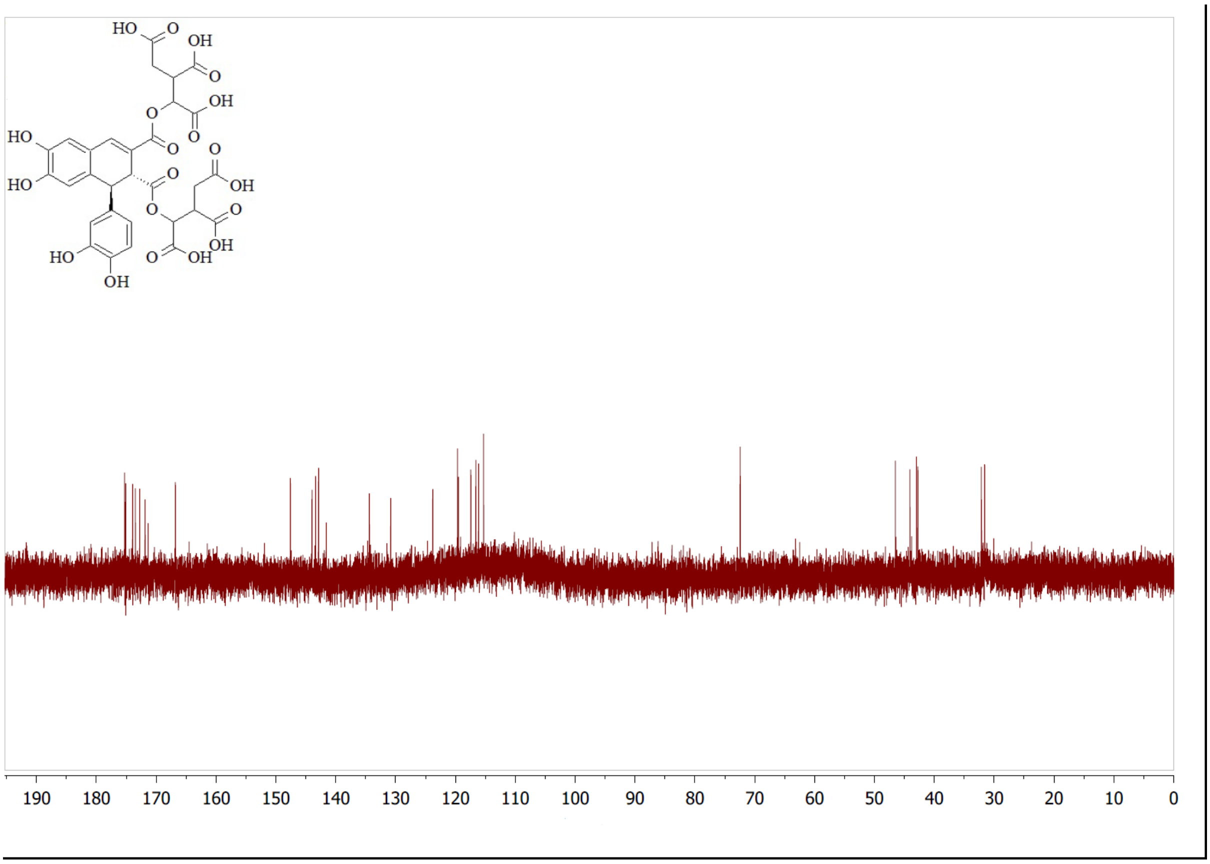

^{1}H and ^{13}C NMR data for **II (s788)** in acetone, 800MHz:

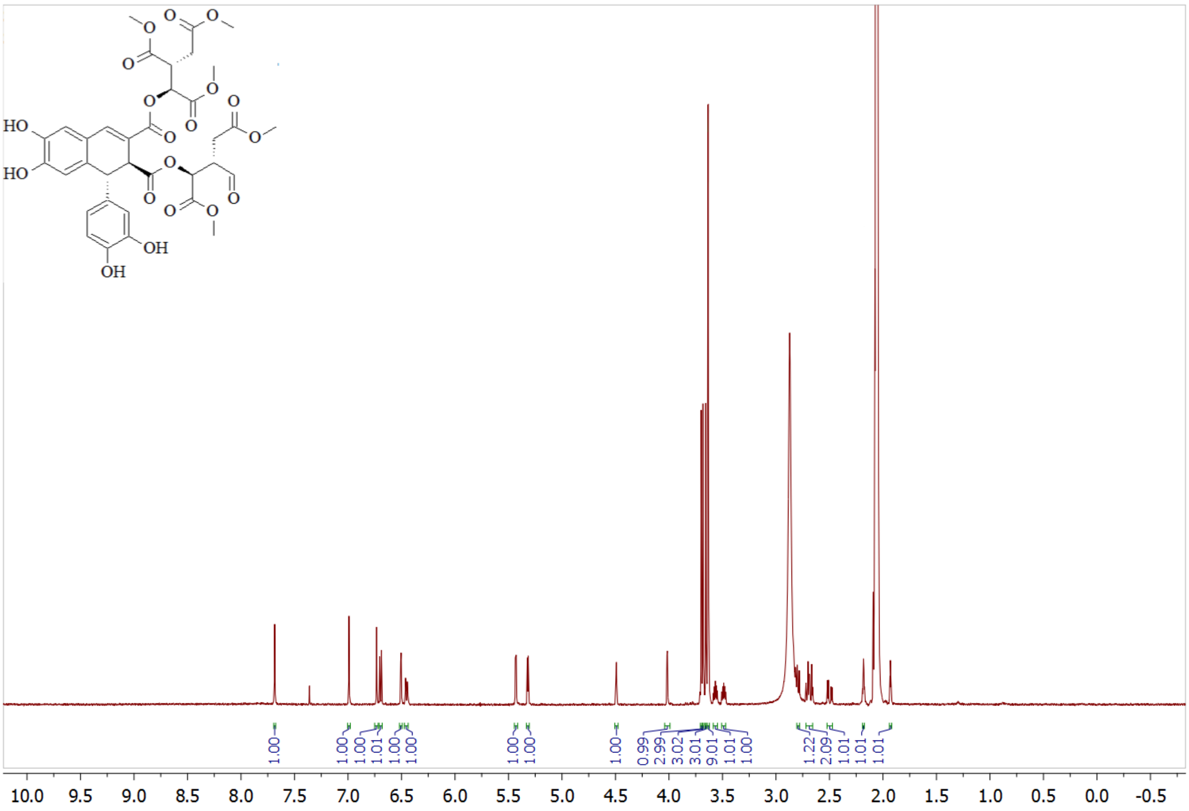

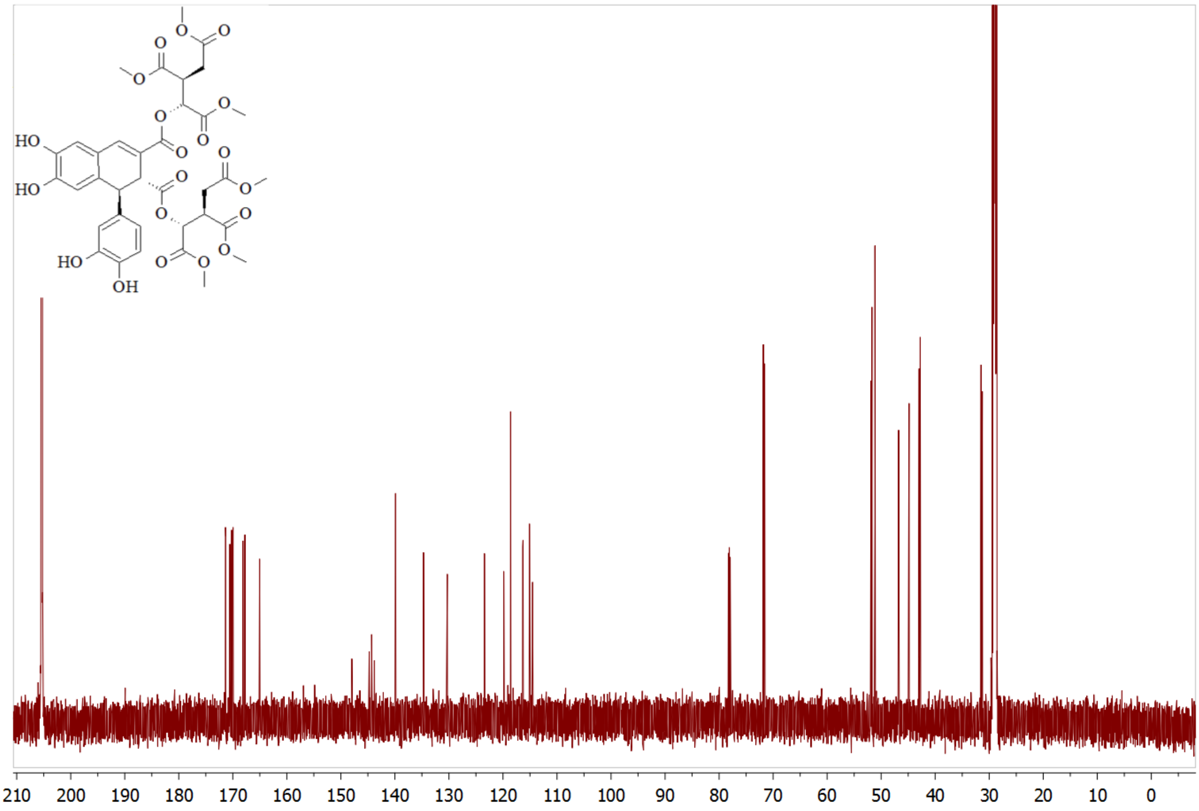

^{1}H and ^{13}C NMR data for **III (s590)** in D_2O, 600MHz:

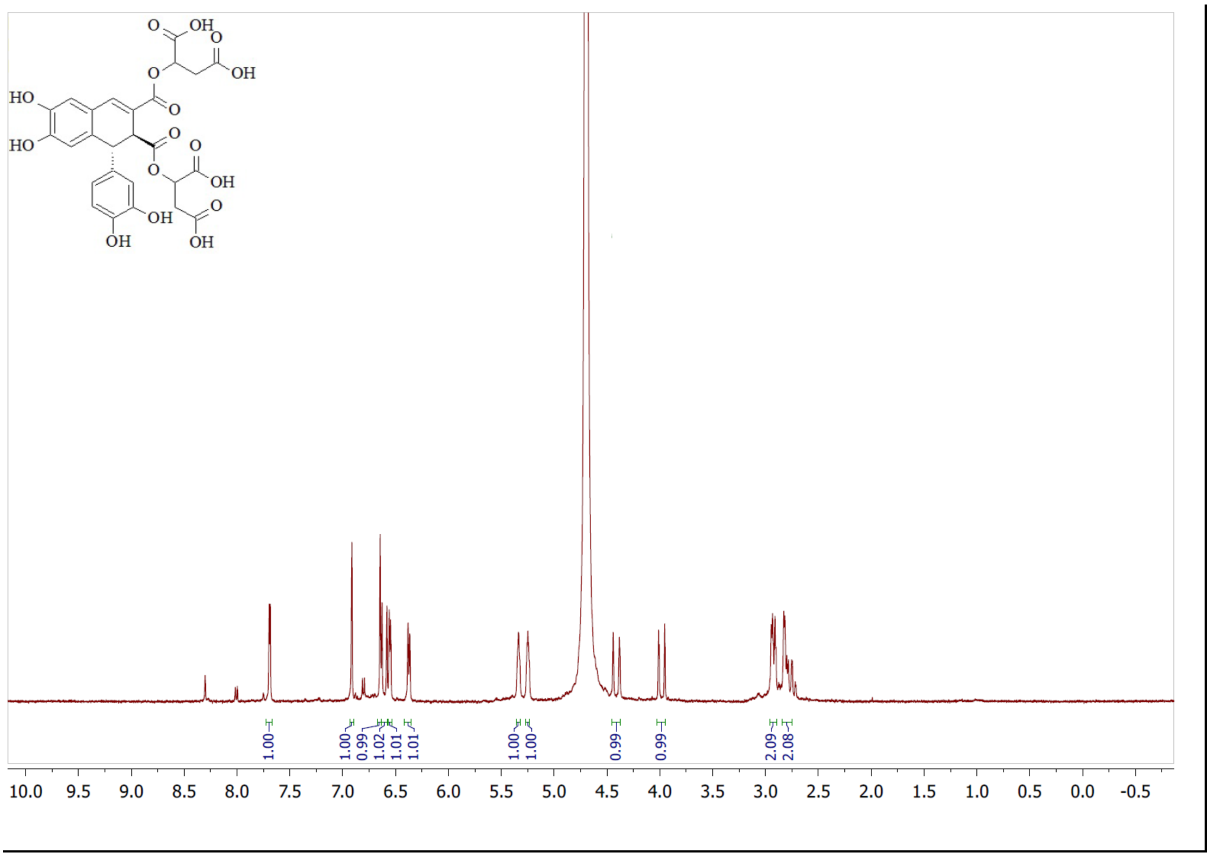

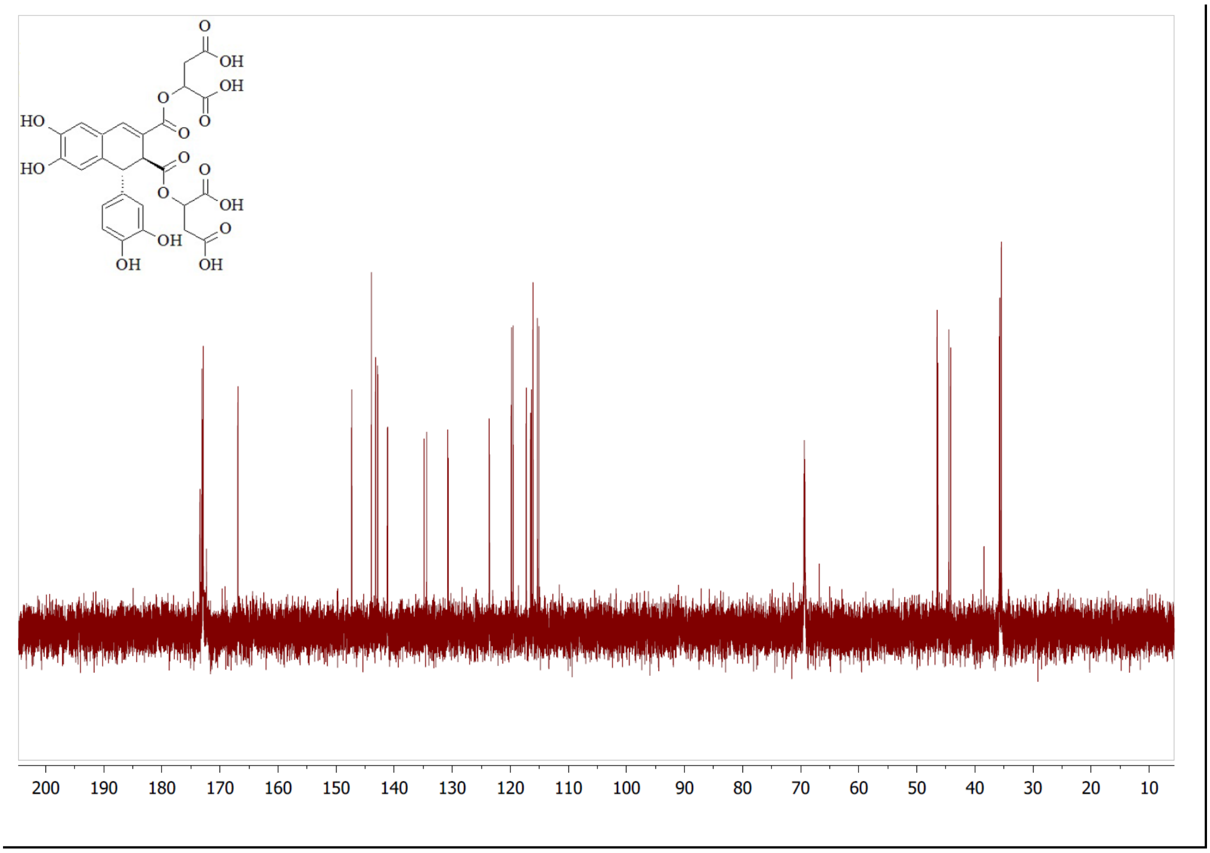

^{1}H and ^{13}C NMR data for **IV (EA)** in D_2O, 600MHz:

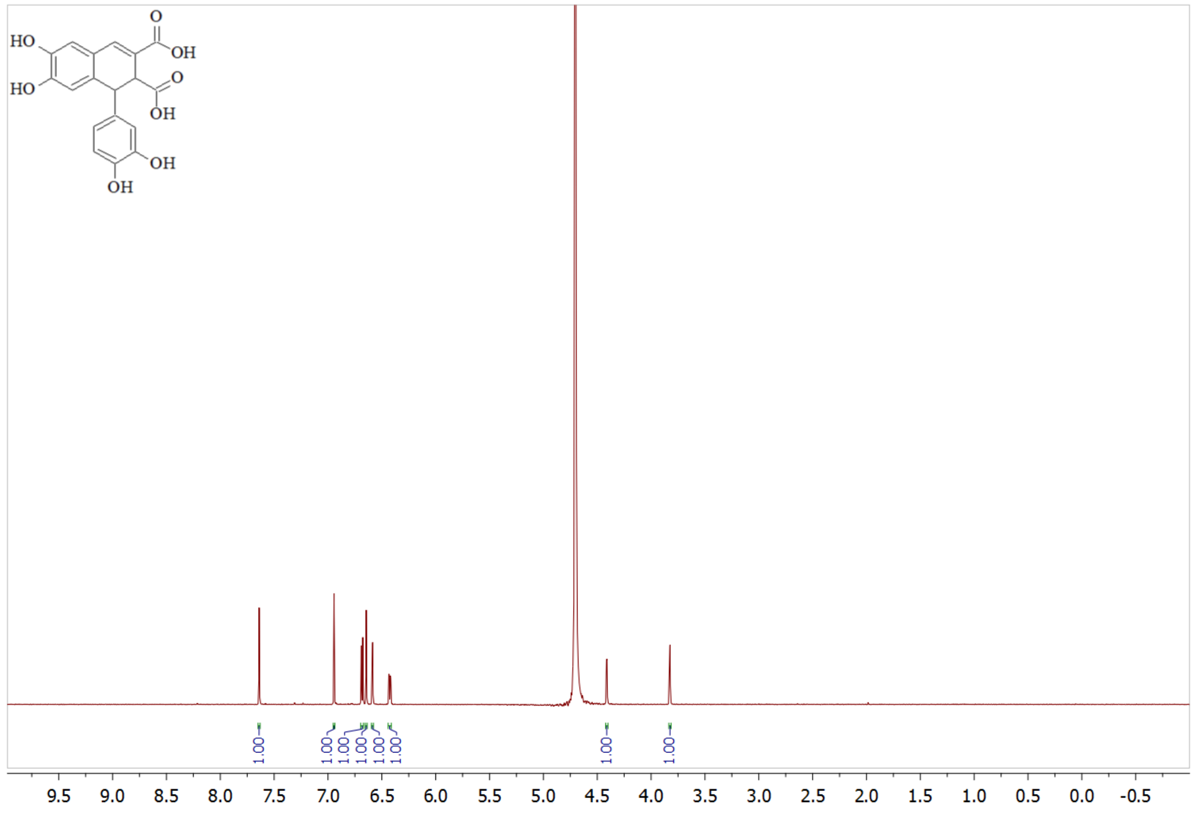

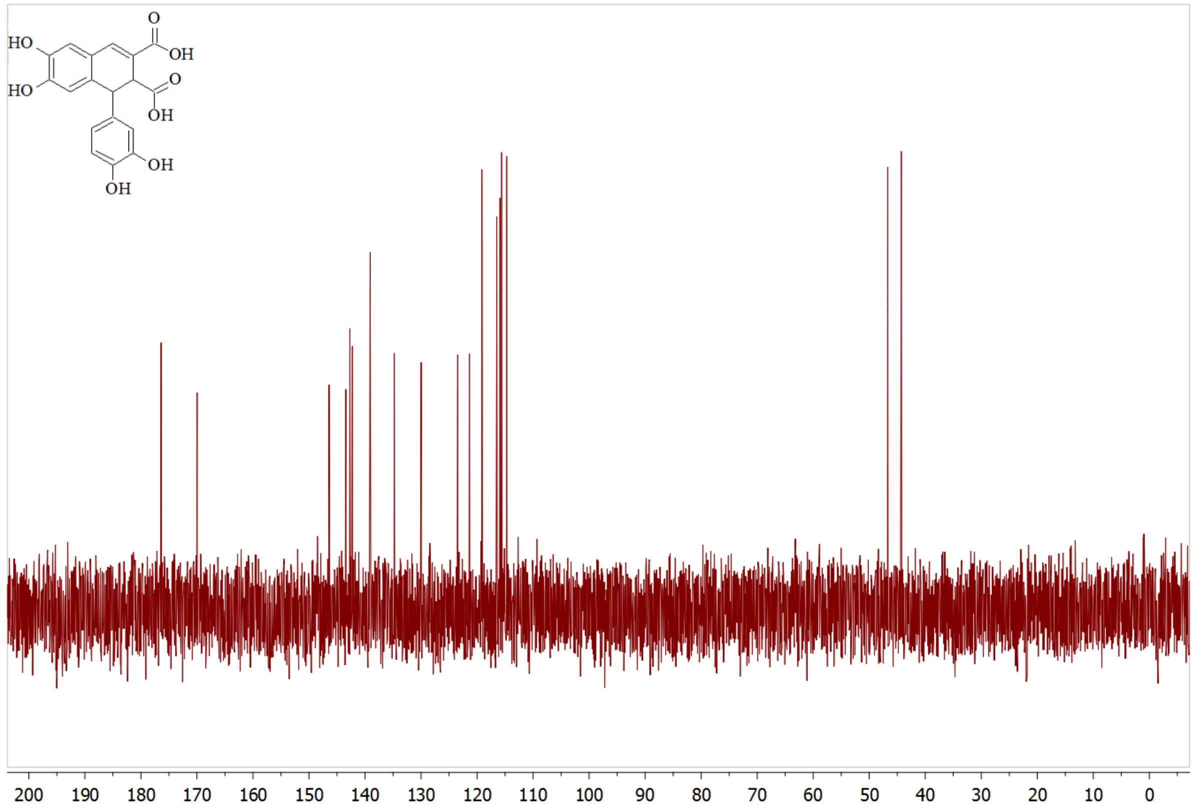

Supplement: Supplementary file 1 [file pharmaceuticals-13-00163-s001.pdf]
